# Characteristics of Human Brain Activity during the Evaluation of Service-to-Service Brand Extension

**DOI:** 10.3389/fnhum.2018.00044

**Published:** 2018-02-09

**Authors:** Taeyang Yang, Seungji Lee, Eunbi Seomoon, Sung-Phil Kim

**Affiliations:** Brain-Computer Interface Laboratory, Department of Human Factors Engineering, Ulsan National Institute of Science and Technology, Ulsan, South Korea

**Keywords:** service brand, brand extension, electroencephalography, event-related potential, neuromarketing

## Abstract

Brand extension is a marketing strategy to apply the previously established brand name into new goods or service. A number of studies have reported the characteristics of human event-related potentials (ERPs) in response to the evaluation of goods-to-goods brand extension. In contrast, human brain responses to the evaluation of service extension are relatively unexplored. The aim of this study was investigating cognitive processes underlying the evaluation of service-to-service brand extension with electroencephalography (EEG). A total of 56 text stimuli composed of service brand name (S1) followed by extended service name (S2) were presented to participants. The EEG of participants was recorded while participants were asked to evaluate whether a given brand extension was acceptable or not. The behavioral results revealed that participants could evaluate brand extension though they had little knowledge about the extended services, indicating the role of brand in the evaluation of the services. Additionally, we developed a method of grouping brand extension stimuli according to the fit levels obtained from behavioral responses, instead of grouping of stimuli *a priori*. The ERP analysis identified three components during the evaluation of brand extension: N2, P300, and N400. No difference in the N2 amplitude was found among the different levels of a fit between S1 and S2. The P300 amplitude for the low level of fit was greater than those for higher levels (*p* < 0.05). The N400 amplitude was more negative for the mid- and high-level fits than the low level. The ERP results of P300 and N400 indicate that the early stage of brain extension evaluation might first detect low-fit brand extension as an improbable target followed by the late stage of the integration of S2 into S1. Along with previous findings, our results demonstrate different cognitive evaluation of service-to-service brand extension from goods-to-goods.

## Introduction

Brand extension refers to a marketing strategy where a well-established brand extends its name to new goods or services (Loken and John, 1993). Since its first introduction in 1960s, brand extension has been widely employed as an effective brand marketing strategy (Gamble, 1967; Tauber, 1988). Brand extension can elevate brand equity by increasing brand loyalty as well as reducing the entry barrier and advertising costs (Tauber, 1981, 1988; Aaker and Keller, 1990), while it can also entail risks that the failure of extension does harm to the well-established parent brand images (Boush and Loken, 1991; Loken and John, 1993; Gürhan-Canli and Maheswaran, 1998; John et al., 1998) and possibly create undesirable associations with the brand in the consumers’ mind (Aaker and Keller, 1990). Therefore, it is important to understand cognitive and behavioral aspects of consumers’ evaluation on brand extension for developing a successful brand extension strategy.

Goods and service are considered as both sides of an important continuum of “offering,” being distinguished from each other by the characteristics such as inseparability, heterogeneity, intangibility, perishability, and a lack of ownership (Zeithaml et al., 1985; Iacobucci, 1998; Lovelock and Gummesson, 2004). Based on this offering level, brand extension can be separated into four types: goods-to-goods, goods-to-service, service-to-service, and service-to-goods (Ramanathan, 2013).

Several behavioral evaluation methods have been proposed to measure the success of extension using explicit survey responses (Völckner and Sattler, 2006; Arslan and Altuna, 2012) or implicit eye tracking movements (Stewart et al., 2004). They showed that a “fit” between a parent brand and extension goods is the most crucial factor for successful brand extension (Aaker and Keller, 1990; Völckner and Sattler, 2006). Therefore, measuring a fit is one of the indicators related to how successful brand extension would be. However, the information provided by the behavioral methods is often limited to account for cognitive processes underlying brand extension evaluation. Alternatively, recent advances in neuroscience have enabled direct measurements of brain activities associated with cognitive processes, providing opportunities to understand cognitive evaluation of brand extension. Hence, using a neuroscience approach, marketers may be able to choose new goods/services with an appropriate fit level for successful brand extension.

A number of neurophysiological studies, mostly using electroencephalography (EEG), have revealed neural activities related to the evaluation of brand extension (Ma et al., 2007, 2008, 2010, 2014a,b; Wang et al., 2012; Jin et al., 2015; Fudali-Czyż et al., 2016; Shang et al., 2017). Recent studies have also investigated the effect of cultural backgrounds on EEG responses to brain extension (Fudali-Czyż et al., 2016), compared EEG patterns between brand extension and new brand creation (Jin et al., 2015) or examined the logo effects on EEG responses to brand extension (Shang et al., 2017). However, all of these studies have focused only on goods-to-goods brand extension, that is, an extension of the product brand name into new goods [e.g., a new beverage, clothes, or an appliance of Coke (Ma et al., 2008)]. Considering that the service industry accounts for an ever-growing share in the global economy (Van Riel et al., 2001), it becomes increasingly important to investigate cognitive processes of consumers dealing with service-related brand extension.

However, not only was there no neuromarketing study on service-related brand extension, but also most marketing studies have focused on goods-to-goods brand extension. Only a few studies have so far examined the cognitive aspects of consumers’ evaluation on service-to-service brand extension (Van Riel et al., 2001; Lei et al., 2004; Brown et al., 2011; Arslan and Altuna, 2012). For instance, Van Riel et al. (2001) suggested a complementarity to the original category as a major cue in evaluating service brand extension. Arslan and Altuna (2012) revealed that service extension is more favorable than goods extension for parent service brand. But, these studies relied on subjective evaluations through surveys, providing only partial information to comprehend consumers’ cognitive evaluation processes on service-related brand extension.

The purpose of this study is, therefore, to conduct the first neuromarketing study to understand cognitive processes for the service-to-service brand extension strategy. To this end, we investigate underlying neural processes using EEG measurements along with the event-related potential (ERP) analysis. The previous neuromarketing studies on goods-to-goods brand extension have revealed that brand extension evaluation was related to several cognitive processes, including conflict monitoring between physical attributes and lexical contents reflected on the ERP component of N2 (N270) (Ma et al., 2007, 2010), and the categorization process reflected on the ERP component of P300 (Ma et al., 2008) and N400 (Wang et al., 2012). Furthermore, an additional study by Ma et al. (2014b) depicted the goods-to-goods brand extension evaluation as a two-stage categorization process expressed in P2 and N400 components. Recently, Fudali-Czyż et al. (2016) conducted a brand extension evaluation study with Indo-European language speakers and showed that N270, P300, and N400 components were responsive to incongruence between the original brand name and extended product name. These studies collectively suggest that some or all of these ERP components would also emerge during the evaluation of service-to-service brand extension.

One of the characteristics that distinguish service from goods offerings is heterogeneity, which refers to a difficulty to support consistent quality for individual consumers (Zeithaml et al., 1985; Iacobucci, 1998). This heterogeneity may lead individuals to recognize larger differences between the parent and extended service offerings compared to the extension of goods brand. Another distinguishable characteristic of the service offering, intangibility (Parasuraman et al., 1985), may make service extension more ambiguous to be systematically categorized than the goods extension. In these regards, we hypothesize that cognitive process engaged in evaluating service-to-service brand extension would not be identical to those in goods-to-goods extension, presumably showing different waveforms of the ERP components compared to those induced by goods-to-goods extension. Benchmarking against the ERP results from the previous goods-to-goods brand extension studies, the present study performs the experiment of service-to-service brand extension and compares experimental ERP results to those of goods-to-goods brand extension. In addition, due to the heterogeneity and intangibility of the service offering, the variation of individual attitudes to each service is generally greater than that to the goods. Consequently, it is challenging to prepare a stimulus pair of the parent brand and extended service representing either similar or dissimilar (i.e., typical or atypical) brand extension, which is different from the case of the previous goods-to-goods brand extension studies where similar or dissimilar brand extension exemplars could be more clearly created by experimenters (Ma et al., 2010; Wang et al., 2012). To overcome this issue, we propose a data-driven method to classify service extension stimulus pairs into similar versus dissimilar groups based on a “fit” level obtained from behavioral responses.

## Materials and Methods

### Participants

A total of 37 participants (19 males, mean age of 22.1 ± 0.33 years old) with normal or corrected-to-normal vision and reportedly no any neurological disorders participated in this study. Smoking and drinking were prohibited within one day before the experiment. All participants provided informed written consent prior to participation according to the approval obtained from the Institutional Review Board of the Ulsan National Institute of Science and Technology (UNISTIRB-16-29-G).

### Experimental Stimuli

Experimental stimuli were collected from previous service-to-service brand extension studies (Lei et al., 2004; Brown et al., 2011; Arslan and Altuna, 2012) and modified for Korean participants (**Table 1**). The set of parent service brand names (S1) consisted of eight service brands from four service categories: e-commerce, finance, airline, and accommodation (two brands per category). It was confirmed that all brands were familiar to participants. The set of extended service names (S2) comprised seven service names per category. Combining all the S1 and S2 data, we created a total of 56 stimulus pairs of S1-S2 service-to-service extension. Due to the aforementioned characteristics of the service offering, it was difficult to determine *a priori* whether each S1-S2 pair in this set was typical or atypical. Instead, we classified each pair based on a “fit” level that was calculated from the participants’ responses obtained in the experiment.

**Table 1:** Stimuli pairs lists.

Category (4)	E-commerce	Finance	Airline	Accommodation
*S1: Service brand name (B1∼B8)*	11st (*B1*)	Kookmin Bank (*B3*)	Korean Air (*B5*)	Lotte Hotel (*B7*)
	G-market (*B2*)	Shinhan Bank (*B4*)	Asiana Airline (*B6*)	Walkerhill Hotel (*B8*)
*S2: Extension service name (ES1∼ES7)*	TV home shopping channel	TV economy channel	Travel agency	Club
	Fashion magazine	Economy magazine	Travel information magazine	Catering service
	Travel agency	Education service	Simultaneous interpretation	Travel information magazine
	Insurance service	Legal counseling	Accommodation reservation	Travel agency
	Marketing consulting	Hospital	TV documentary channel	Legal counseling
	Newspaper	Simultaneous interpretation	Rent-a-car	Hospital
	Food delivery	Airline	Education	Finance

*A total of 56 stimuli pairs were written as following examples: TV home shopping channel of 11st (*B1-ES1*), Simultaneous interpretation service of Shinhan Bank (*B4-ES6*), or Legal counseling of Walkerhill Hotel (*B8-ES6*).*

### Experimental Procedure

**FIGURE 1:**
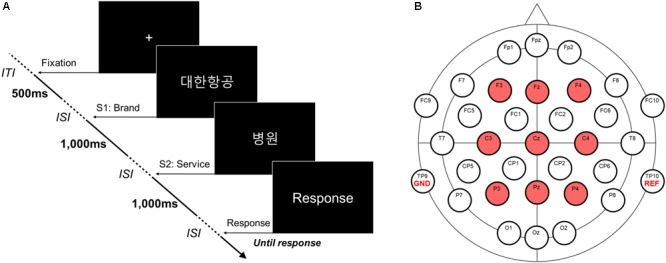
An experimental task and an EEG montage. **(A)** An experimental task for evaluating service brand extension. A total of 56 S1-S2 pairs were presented consecutively in Korean [e.g., Hospital service 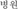 of Korean airline 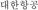. There was no time limitation for response. Inter-trial interval (*ITI*) and inter-stimulus interval (*ISI*) were 2,000 and 500 ms, respectively. **(B)** EEG montage. A total of 32 EEG channels, including one reference channel (i.e., TP10) and one ground channel (i.e., TP9) were simultaneously measured. Nine channels (i.e., F3, Fz, F4, C3, Cz, C4, P3, Pz, and P4) were used to analyze ERPs.

Participants were seated in a dim and electrically shielded room. Before the experiment, each participant was given a written instruction about the experiment. The experiment consisted of one training block followed by four test blocks, each containing 56 trials. In a single block, each of the 56 S1-S2 stimulus pairs was randomly presented to participants. All the visual stimuli were presented on a 27-inch monitor (QH2700-IPSMS, Achieva Korea, Incheon, South Korea) positioned at 60 cm distant from participants’ eyes. The S1-S2 presentation paradigm with an explicit evaluation task (Ma et al., 2007, 2008; Jin et al., 2015; Fudali-Czyż et al., 2016; Shang et al., 2017) was employed in this study (**Figure 1A**). At the beginning of each trial, a fixation (i.e., white cross) appeared for 500 ms at the center of the black screen. Immediately after the fixation disappeared, one of the service brand names (S1) was presented, followed by the presentation of one of the extended service names (S2). Each stimulus was displayed for 1,000 ms with an inter-stimulus interval (*ISI*) of 500 ms. After the presentation of S2, participants were asked to evaluate whether the given brand extension was acceptable or not to themselves with a keyboard (“right arrow” key for acceptable and “left arrow” key for not acceptable). The next trial began 2,000 ms after participants responded. Participants were instructed to take a break long enough between the blocks.

After the experiment, participants took a memory test that was used to verify that they attended to the experiment. The memory test consisted of 30 stimulus pairs, including 11 brand extension pairs not used in the experiment. In the memory test, participants answered whether they had seen a given brand extension pair during the experiment. All participants showed a tolerable error rate under 33% (i.e., less than 10 wrong answers to 30 questions). Finally, participants filled in the survey form that consisted of the seven questions asked for each S1-S2 pair: (Q1) acceptance rate; (Q2) quality expectancy; (Q3) preference; (Q4) similarity between a typical service of S1 brand and S2 service; (Q5) acceptance rate of the brand extension strategy (i.e., attitude toward the marketing strategy); (Q6) attitude toward the S1 brand; and (Q7) own knowledge level regarding the S2 service (see Supplementary Material for the questions). Participant was asked to indicate how they agreed with each question with the 7-point Likert scale (1 – strongly disagree, 7 – strongly agree).

### EEG Recordings

During the experiment, scalp EEG signals were recorded (band-pass filtering: 0.05-100 Hz, sampling rate: 500 Hz) using the 31-channel wet-electrode EEG recording system (actiCHamp, Brain products GmbH, Gilching, Germany) from the following electrode locations: FP1, FPz, FP2, F7, F3, Fz, F4, F8, FC9, FC5, FC1, FC2, FC6, FC10, T7, C3, Cz, C4, T8, CP5, CP1, CP2, CP6, P7, P3, Pz, P4, P8, O1, Oz, and O2 (in accordance with the International 10/20 system). We distributed electrodes over the wide range of scalp from prefrontal to occipital areas in a sagittal direction and symmetrically in the coronal direction, using the maximum number of EEG channels provided by the EEG apparatus. An additional electrode was applied to the left mastoid (TP9) as a ground. The EEG signals were on-line referenced to the right mastoid (TP10) (**Figure 1B**). Impedance of every electrode was set below 10 kΩ during the recordings.

### Data Analysis

The behavioral data obtained in the experiment included affirmative responses during the experiment and responses to the seven questions after the experiment. The affirmative rate (AR) of each of the 56 pairs was calculated by averaging 4 binary acceptance responses. Note that among 37 participants, the data of a participant whose experiment was interrupted and 4 participants who did not response to survey correctly were excluded from the analysis. In addition, the data of 13 participants were additionally excluded from the EEG analysis due to following issues: (1) the EEG data of 4 participants were visually inspected as too much noisy despite the independent component analysis (ICA) method to reduce artifacts; and (2) the AR data of 9 participants failed to give the minimum number of trials for each fit group (the minimum of 12). Consequently, the EEG and behavioral data of a total of 19 participants were analyzed (9 males, mean age of 20.6 ± 0.48 years old).

**FIGURE 2:**
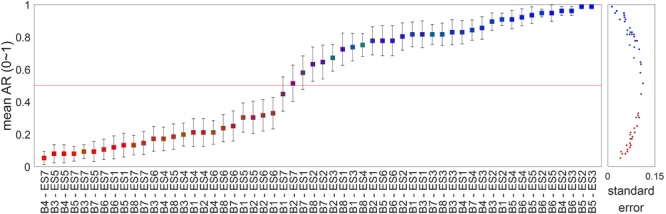
The distribution of the affirmative rate (AR) for each stimulus pair of service-to-service extension. Each squared point shows the mean AR across participants in response to each pair of brand name (S1) and extended service (S2) stimuli (a total of 56 stimuli) (see the section “Data Analysis”). The black lines across the squares denote the SEM AR. These values of the SE are also illustrated in the right panel as dotted points. The RGB colors of the squares and dots represent the frequency that participants assigned a pair to each of the three fit groups: low- (red), mid- (green), and high-fit (blue).

To investigate behavioral responses of “fit” between S1 and S2 stimuli, each S1-S2 pair was assigned to a low-fit (AR = 0), mid-fit (AR = 0.25, 0.5, or 0.75), or high-fit (AR = 1) group depending on each participant’s subjective AR response. Specifically, as individual participants were likely to evaluate the same S1-S2 pair differently, the grouping of the stimulus pairs was formed individually for each subject. **Figure 2** shows the mean and SE of AR across participants. In **Figure 2**, we also visualized the number of times each stimuli pair was assigned to each group via color-coding (i.e., The RGB value of each point represents the ratio of low-, mid-, and high-fit, respectively). **Figure 2** shows that the variance of AR increased for the S1-S2 pairs with moderate average near 0.5 and that some pairs were perceived to suit to a subset of participants but not to others (e.g., those in purple). This indicates that the perceived fit levels for certain brand extension substantially varied across individuals and thus supports our approach of individual grouping of stimuli. We also compared the reaction time (RT) and responses to seven questions between the three groups using a one-way repeated measure ANOVA with a Bonferroni’s corrected *post hoc* paired *t*-test.

As a further analysis for behavioral data, to examine relationships between behavioral responses, a pairwise Pearson’s correlation analysis was conducted between every combination of eight responses, creating an 8 × 8 symmetric correlation coefficient matrix per subject. To examine whether a correlation between a particular pair of responses was statistically different from a correlation between another pair, we conducted a pairwise comparison between every possible pair of the correlation coefficients using a paired *t*-test with Bonferroni’s correction (*N* = 19). For this test, each correlation coefficient (*r*) was transformed to a *z*-value using the Fisher’s *z*-transformation (Eq. 1), because the correlation coefficient was limited in the range of (-1, 1) resulting in the violation of normality.

(1) $$\begin{array}{c} z=\frac{1}{2}\ln\left(\frac{1+r}{1-r}\right) \end{array}$$

The recorded EEG data were analyzed offline using the MATLAB software (version 2016a, MathWorks, Inc., MA, United States). Among the 31 channels, the following 9 channels were selected for the analysis to be comparable with the previous results (Ma et al., 2014b; Fudali-Czyż et al., 2016): F3, Fz, F4, C3, Cz, C4, P3, Pz, and P4. Although other fronto-central (FC9, FC5, FC1, FC2, FC6, and FC10) and centro-parietal (CP5, CP1, CP2, and CP6) channels were additionally analyzed in the previous studies, we did not include them in the analysis because our EEG system did not provide midline channels needed to compare the results. The EEG signals at each channel were band-pass filtered with 0.5 and 50 Hz cutoff frequencies using a FIR filter. Next, eye blink artifacts were removed using the ICA method. Then, EEG epochs were extracted from a 1,000-ms data segment (-200∼800 ms post-stimulus) time-locked to the onset of the second stimulus (S2) and corrected to each baseline (-200∼0 ms time-locked to the onset of S2). The EEG epochs from the trials of interest (e.g., the trials of S1-S2 pairs leading to a high-fit response) were averaged to obtain ERPs, excluding any trial with a peak-to-peak deflection exceeding ± 85 μV. Finally, the ERP waveforms were low-pass filtered using a Butterworth filter (30 Hz, third order zero-phase IIR filter).

To statistically assess the ERP components observed during the evaluation of service-to-service brand extension, 3 (Fit) × *K* (Channel) two-way repeated measures ANOVA tests were applied to the amplitudes of the observed ERP components. The number of channels, *K*, was determined depending on the observation of ERP components. In other words, if the tested ERP component was visually pronounced only in a subset of the nine channels, *K* was the number of those channels showing the tested ERP components. For *post hoc* tests on the main effect of the factor of fits, Bonferroni’s corrected pairwise *t*-tests with the ERP component amplitude data were conducted between three fit levels (low-, mid-, and high-). For *post hoc* tests on the interaction effect of the two factors, one-way repeated measures ANOVA tests with the ERP component amplitude data for *K* channels were conducted. Greenhouse-Geisser correction was used when Mauchly’s test showed that the sphericity assumption was violated (in this case, uncorrected degrees of freedom were reported as 𝜀 in addition to a corrected *p*-value). Bonferroni’s correction was used for adjusting *p*-values for multiple comparisons.

## Results

### Behavior Results

**FIGURE 3:**
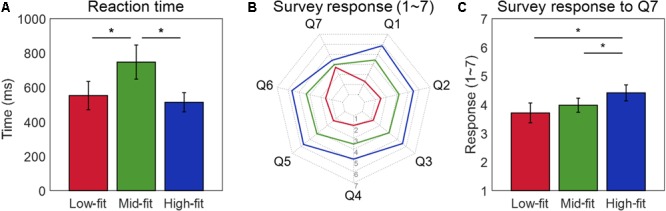
Comparison of behavioral responses between three fit groups. **(A)** Group-wise reaction time of participants when evaluating whether a given brand extension was acceptable or not to themselves by pressing a keyboard. **(B)** Group-wise survey responses for each of seven questions. All pairwise *t*-tests for the six questions (Q1–Q6), except for Q7, showed significant difference between three groups. **(C)** Group-wise survey responses for Q7 regarding participants’ background knowledge about extended service. ^∗^Indicates a significant difference between two fit groups (*p* < 0.05).

The ANOVA showed that reaction time (RT) was significantly different among the three fit groups (*F*_(2,36)_ = 15.416, *p* < 0.001) (**Figure 3A**). A Bonferroni’s corrected multiple comparison *post hoc* test revealed that RT of the mid-fit group [mean (*M*) = 747.529 ms, SE = 99.186] was significantly slower than those of the high-fit (*M* = 513.841 ms, SE = 55.601, *t*_(18)_ = 4.220, *p* < 0.001) and the low-fit (*M* = 552.773, SE = 82.345, *t*_(18)_ = 4.467, *p* < 0.001) groups, while no significant difference was found in RT between the high-fit and low-fit groups (*t*_(18)_ = 1.109, *p* = 0.282). Repeated measures ANOVA for subjective responses to seven questions revealed significant differences among the three fit groups for every question (*ps* < 0.05) (**Figure 3B**). Bonferroni’s corrected pairwise *post hoc* t-tests between the fit groups showed more positive subjective responses in the high-fit group than mid-fit group, and subsequently in the mid-fit group than low-fit group, for every question except for Q7 (i.e., prior knowledge about an extended service) in which no difference was found between the low- and mid-fit groups (*t*_(18)_ = 1.431, *p* = 0.170) (**Figure 3C**).

**FIGURE 4:**
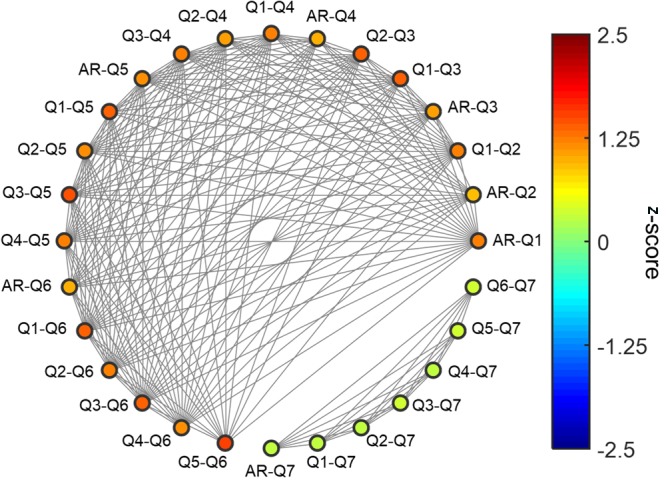
A diagram of 28 correlation coefficients between eight questions. A total of 28 correlation coefficients of behavioral responses between every possible pair of eight questions (seven questions and the AR) were represented as the nodes (colored circles). Each node was color-coded using the *z*-score of the correlation coefficients. A pair of nodes was connected by gray lines if there were no significant differences between the correlation coefficients of the nodes (*z*-scores, *p* < 0.05).

The pairwise Pearson’s correlation coefficients between eight behavioral responses (seven responses to survey questions and one AR response during the task) were calculated and transformed to Fisher’s *z*-values. The resulting 28 (_8_C_2_ = 28) *z*-values were illustrated as nodes in **Figure 4**. The pairwise *t*-test for identifying differences between the *z*-values of a pair of nodes showed that the *z*-values associated with the seventh survey question (Q7: own knowledge level regarding the S2 service) were significantly lower than other *z*-values (*p* < 0.05). Note that there was no significant difference within the Q7-related *z*-values (*p* > 0.05), indicating that Q7 was not correlated with any other behavioral responses.

### ERP Results

**FIGURE 5:**
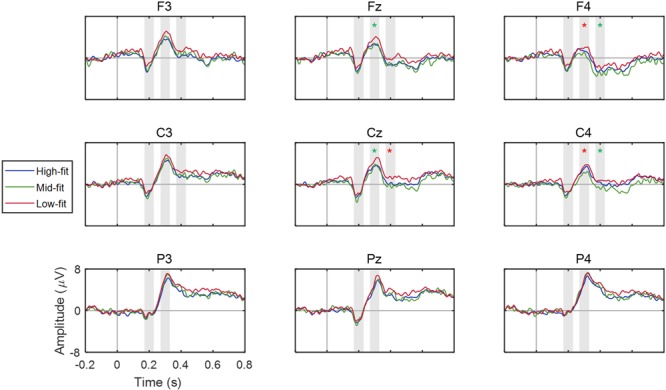
ERP waveforms of nine EEG channel locations. The mean ERP waveforms across participants were obtained for each fit group and each channel. The vertical bars at 0 s denote the onset of stimulus (extended service; S2). Red and green asterisk marks (^∗^) indicate a significant difference (*p* < 0.05) and a trend of difference (*p* < 0.10) between low and high fit groups, respectively. The shaded areas represent the ERP components observed in this study.

Although our experimental paradigm was identical to those in the previous goods-to-goods brand extension studies (Ma et al., 2007, 2008, 2014b; Fudali-Czyż et al., 2016; Shang et al., 2017), the ERP waveforms of our study exhibited differences from the previous ones. In our study, the ERPs showed that three positive or negative peaks—N2 (170∼230 ms), P3 (270∼330 ms), and N400 (370∼430 ms)—were prominent in every stimulus group (**Figure 5**). N2 and P3 were observed at all electrodes (F3, Fz, F4, C3, Cz, C4, P3, Pz, and P4) in the frontal, central, and parietal areas. N400 was observed at six electrodes (F3, Fz, F4, C3, Cz, and C4) in the frontal and central areas, but not in the parietal area.

**FIGURE 6:**
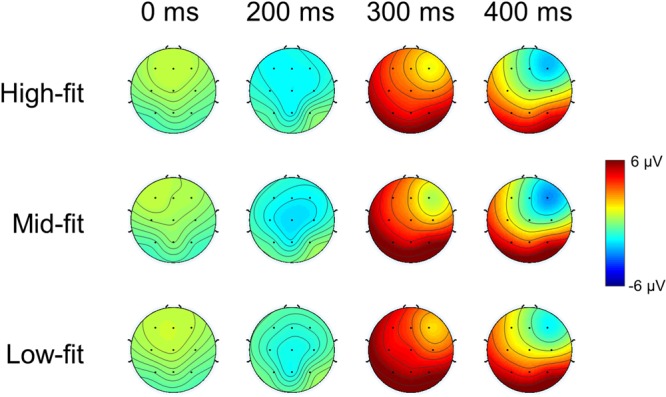
ERP topography. The topographies of the ERP amplitudes for three different fit groups (i.e., high-, mid-, and low-fit) at 0, 200, 300, and 400 ms after stimulus (extended service; S2) onset. The color level denotes the mean ERP amplitude across participants. The black dots represent the EEG channels from which ERPs were analyzed in this study: F3, Fz, F4, C3, Cz, C4, P3, Pz, and P4.

As such, we further investigated the spatial patterns of these ERP components by constructing topographies of the ERP amplitudes for three different fit groups (i.e., high-, mid-, and low-fit stimuli) at 200, 300, and 400 ms after stimulus onset. The topographies at these three latencies showed that the low-fit stimuli elicited larger positive amplitudes at 300 ms as well as smaller negative amplitudes at 400 ms compared to other stimuli (**Figure 6**). At both 300 ms and 400 ms, the amplitudes tended to increase following the sagittal direction from anterior to posterior regions. The most salient spatial pattern was relatively smaller amplitudes at right frontocentral areas at both 300 and 400 ms.

**FIGURE 7:**
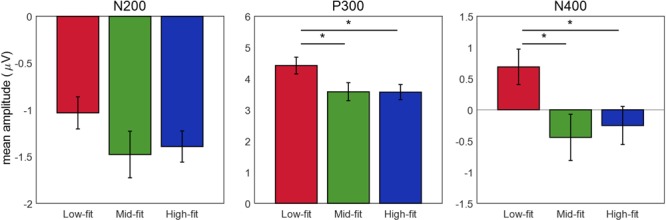
Comparison of the ERP component amplitudes among the fit groups. The mean amplitudes of three ERP components across participants and channels, including N200, P300, and N400, were calculated for each fit group (low-, mid-, and high-fit). ^∗^Indicates a significant difference between two fit groups (*p* < 0.05).

To quantify these observations, the statistical tests (see the section “Data Analysis”) on the amplitudes of each of the three components were conducted individually (**Figure 7**). For the N2 amplitude, two-way repeated measure ANOVA showed neither interaction nor any significant main effects of both factors (Fit and Channel) (*p* > 0.05).

For the P300 amplitude, two-way repeated measure ANOVA revealed a trend of main effect of “Fit” (*F*_(2,36)_ = 2.472, *p* = 0.0986) and a significant main effect of “Channel” (*F*_(8,144)_ = 22.302, 𝜀 = 0.357, *p* < 0.001). *Post hoc* tests on the main effect of “Fit” revealed that the P300 amplitude was significantly higher for the low-fit group (*M* = 4.420 μV, SE = 0.0205) than the high-fit (*M* = 3.567 μV, SE = 0.0186, *t*_(170)_ = 4.776, *p* < 0.001) or the mid-fit (*M* = 3.579 μV, SE = 0.0221, *t*_(170)_ = 5.709, *p* < 0.001) groups. The difference in the P300 amplitude between the high-fit and mid-fit groups was insignificant (*t*_(170)_ = 0.0611, *p* = 0.951). In addition, ANOVA showed a significant interaction effect between the “Fit” and “Channel” factors on the P300 amplitude (*F*_(16,288)_ = 2.207, 𝜀 = 0.367, *p* = 0.049). Further analyses on the interaction effect revealed significant effects of “Fit” only at F4 (*F*_(2,36)_ = 4.937, *p* < 0.05) and C4 (*F*_(2,36)_ = 4.470, *p* < 0.05) over the right hemisphere. The result also showed a trend of significant effects at the Fz (*F*_(2,36)_ = 2.861, *p* = 0.0703) and Cz (*F*_(2,36)_ = 3.047, *p* = 0.06) over the midline.

For the N400 amplitude, two-way repeated measure ANOVA showed a significant main effect of “Channel” (*F*_(5,90)_ = 28.97, 𝜀 = 0.508, *p* < 0.001) and a trend of main effect of “Fit” (*F*_(2,36)_ = 2.475, *p* = 0.0983). *Post hoc* tests on the main effect of “Fit” showed that the N400 amplitude was significantly more negative for the high-fit (*M* = -0.252 μV, SE = 0.0286, *t*_(113)_ = 4.490, *p* < 0.001) and the mid-fit (*M* = -0.442 μV, SE = 0.0347, *t*_(113)_ = 4.266, *p* < 0.001) groups than the low-fit group (*M* = 0.688 μV, SE = 0.0267). The difference of the N400 amplitude between the high-fit and mid-fit groups was insignificant (*t*_(113)_ = 0.745, *p* = 0.458). The test also showed a trend of an interaction effect between the “Fit” and “Channel” (*F*_(10,180)_ = 1.954, 𝜀 = 0.494, *p* = 0.0942). Further analyses on the interaction effect revealed significant effects of “Fit” only at Cz (*F*_(2,36)_ = 3.373, *p* < 0.05). The result also showed a trend of significant effects at F4 (*F*_(2,36)_ = 3.0702, *p* = 0.0587) and C4 (*F*_(2,36)_ = 3.149, *p* = 0.0549) over the right hemisphere.

## Discussion

Previous studies on neural correlates of the brand extension evaluation using ERP methods considered only goods-to-goods brand extension. On the contrary, this ERP study investigated neural activities during service-to-service brand extension.

Except for the difference of the type of extension, namely service versus goods, a crucial difference between the current study and the previous brand extension studies was a way of grouping stimuli. In the previous studies (Ma et al., 2007, 2008, 2010, 2014a,b; Wang et al., 2012; Jin et al., 2015; Fudali-Czyż et al., 2016), stimulus pairs were grouped into high-fit, mid-fit, and low-fit sets manually by experimenters based on the product category each goods belongs to (e.g., beverage versus non-beverage, snacks, clothing, or household appliances). It reflects an implicit assumption that there is little across-subject variation in perceived fit levels for a given goods-to-goods pair. However, such an assumption may not work well with service offering because it is too ambiguous to categorize services in accordance with a common sense and the experience of the quality of services can considerably vary over consumers, having each consumer mentalizing different concepts of the same service. Hence, for a service-to-service brand extension study, stimulus pairs may need to be grouped based on subjectively felt fit levels. In our study, stimulus pairs were divided into three groups (i.e., low-, mid-, and high-fit) according to participants’ AR responses during the brand extension evaluation task. Our results showed large variance across participants in the ARs for ambiguous stimuli, supporting the idea that stimulus grouping should be conducted based on subjective responses (**Figure 2**).

Some examples of service-to-service extension showed interesting grouping results. For instance, the education service extended off two airline service brands (i.e., Korean Air and Asiana Airline) was categorized as high-fit stimuli by 18 and 15 participants (out of 19), respectively. Also, 14 participants evaluated interpretation service extended off two financial service brands (i.e., Kookmin Bank and Shinhan Bank) as high-fit brand extension. In most cases, the extension of two brands belonging to the same service category (e.g., Airline) to a certain service was evaluated as a similar fit level. Exceptionally, participants’ evaluations on the brand extension of two brands of the hotel category were slightly different. These results might indicate that consumers largely consider the characteristics of service category first and then fine-tune their evaluation based on the characteristics of individual brands on occasion.

The result of RT in our study was similar to that of the previous study carried by Ma et al. (2007), which compared beverage brand extension to four product categories (i.e., beverage, snack, clothing, and household appliances). In their study, RT was the fastest for beverage-beverage (high-fit), the slowest for beverage-snack (low-fit) and in between for other cases (mid-fit). It indicates that our S1-S2 grouping based on participants’ responses could provide a valid means to estimate a fit level during service-to-service brand extension evaluation. Yet, no difference in RT was found between the low-fit and high-fit stimuli in our data, dissimilar to the previous results about goods-to-goods brand extension where fit levels perceived by consumers could be inferred from the RT data (Ma et al., 2007, 2008; Jin et al., 2015). In the case of service-to-service brand extension, however, we found that RT for both high-fit and low-fit stimuli was indistinguishable, which may imply that more than just RT data are needed to explore why a consumer evaluated various cases of brand extension with different fit levels. This leads us into exploring neural activity to find distinguishable responses between high-fit and low-fit stimuli.

Our behavioral data analysis showed that the knowledge level of extended services was not correlated with other behavioral responses associated with brand extension evaluation such as AR, expected quality, preference, and brand attitude. In the brand extension evaluation task of this study, the brand name was a unique cue with which participants evaluated brand extension. Therefore, our results indicate that behavioral responses to an unfamiliar service of a familiar brand were primarily affected by participants’ experiences to that brand name (S1) even though they did not know well about extended service (S2). It may underscore a key role of brand images in brand extension evaluation.

The ERP components resulted from our study can be compared with the previous results from the goods-to-goods brand extension evaluation. Compared to the goods-to-goods brand extension study by Ma et al. (2014b) where P2 and N400 were reportedly observed, our ERP analysis results showed only N400. On the other hand, the result of another goods-to-goods brand extension study by Fudali-Czyż et al. (2016) exhibited N2 and P300 similar to our results. A primary difference between these two goods-to-goods extension studies was the way participants responded during the experimental task. In the study by Ma et al. (2014b), participants did not have to behaviorally respond, whereas in the study by Fudali-Czyż et al. (2016), participants explicitly responded whether each brand extension pair was affirmative or not. Wang et al. (2012) suggested that P300 could reflect a combined effect of categorization and explicit evaluation during the brand extension evaluation task as they did not observe P300 in their implicit task. Since our task also required explicit responses by participants and elicited the ERP components similar to those by Fudali-Czyż et al. (2016), our results may support the suggestion of Wang et al. (2012) and Fudali-Czyż et al. (2016) that explicit evaluation of brand extension may involve categorization manifested by the ERP components of N2 (N270), P300, and N400. Our results of the pronounced P300 with the maximum at parietal areas and N400 with the maximum at frontal areas were also consistent with to the previous results (Wang et al., 2012; Ma et al., 2014b).

However, our results differed from those by Fudali-Czyż et al. (2016) or Ma et al. (2008) with respect to the P300 amplitude. The P300 amplitude was higher for the low-fit (incongruent) group than others in our study whereas it was higher for the high-fit (congruent) group in the previous studies. In the previous studies, participants responded to each stimulus pair in terms of approvability (i.e., “This brand extension fits well enough to sound approvable to me.”). However, in our study, participants were likely to evaluate each stimulus pair in terms of improbability (i.e., “This brand extension is highly improbable”). As mentioned above, consumers may feel more difficult in categorizing service than goods. Therefore, it might be challenging to them to divide stimulus pairs based on approvability. Instead, they might evaluate how improbable a pair was, classifying the pair into the low-fit (improbable) group versus others. Our ERP result supports this conjecture regarding a difference of evaluation processes between goods-to-goods and service-to-service brand extension. Previous studies about P300 amplitude showed that P300 amplitude is relevant to detection of improbable target stimuli (Duncan-Johnson and Donchin, 1982; Stuss et al., 1986; Azizian et al., 2006). Hence, a higher P300 amplitude for the low-fit group in our results might indicates the detection of an improbable (low-fit) stimulus pair.

Our results of N400 might represent the later cognitive process of brand extension evaluation. N400 was considered to reflect a late categorization process according to the integrality category concept between S1 and S2 (Ma et al., 2014b). In the previous study (Ma et al., 2008), N400 was predominantly evoked by the conflict condition (i.e., non-beverage products of beverage brand) and noted that it could be an endogenous index for the evaluation of unfitted brand extension. Another study by Wang et al. (2012) reported stronger N400 at frontal areas and suggested that it reflected the integration and conceptual analysis process of the extended goods into the parent brand. In the present study, we observed that the N400 amplitude for the low-fit stimulus group was greater than zero with a negative peak (**Figure 7**). We speculated that this weak N400 component occurred as participants filtered out the low-fit stimulus pairs in terms of improbability (reflected by strong P300), skipping subsequent integration and categorization process represented by N400. On the contrary, for the mid-fit and high-fit stimulus pairs, participants might execute the late integration and categorization process to evaluate service-to-service brand extension more precisely. Therefore, N400 amplitude for mid-fit stimuli was more negative than high fit stimuli, showing more incongruence.

In our ERP data, N400 appeared predominantly at frontal channels, showing more negative amplitudes over right frontal areas (**Figure 6**). The study by Wang et al. (2012) reported stronger N400 at frontal areas and suggested that it reflected the integration and conceptual analysis process of the extended goods into the parent brand. Similarly, the observation of frontal N400 in our study may reflect the integration of the extended service with the parent brand. In addition, Stringaris et al. (2006) suggested that the neural activation in the right frontal cortex indicate the attempt to establish a semantic relationship between successive items. Therefore, our results demonstrated more negative N400 amplitudes for high-fit and mid-fit stimuli than for low-fit stimuli, indicating that the establishment of a semantic relationship of brand and service might be facilitated for the stimuli with higher fit levels. Taking into consideration the implication of P300 for the detection of improbable low-fit stimuli, we speculate that there may be a “threshold” to detect low-fit stimuli based on improbability and then evaluate the rest stimuli through a process of integrating and establishing the semantic relationship between parent brand and extended service.

Ma et al. (2014b) explained about goods-to-goods brand extension as the two-stage cognitive process; consumers initially categorize a stimuli pair according to physical similarity followed by an analytic categorization process. However, our results may suggest that consumers are likely to evaluate service-to-service brand extension with a different categorization process. An *anterior* N2 component was observed at frontal and central areas in the present study, but no significant difference in the N2 amplitude was found between different fit levels. Previous studies revealed that *anterior* N2 is elicited in the sequential matching task to discriminate whether the physical attributes (i.e., color or shape) are equal between sequentially presented stimuli (Wang et al., 2003, 2004; Folstein and Van Petten, 2008; Luck, 2014). Therefore, no difference in *anterior* N2 amplitude between different fit levels in our study may indicate that consumers did not evaluate physical aspects of S1 and S2, because of the intangibility of service offering (Iacobucci, 1998).

There are some limitations in our study. First, the number of mid-fit stimuli turned out to be relatively small compared to other stimulus groups. Even with the exclusion of some participants’ data by setting the minimum number of mid-fit stimuli, we could not balance the number of mid-fit stimuli with those of other groups. It may be due to that it was more natural to categorize service offering as binary classes (i.e., low or hard-fit). Second, categorization of the fit levels of service-to-service brand extension may be more complex than simple three levels of high-, mid-, and low-fit groups. Perhaps, a simple evaluation of brand extension with a single “fit” parameter might not be suitable for service-to-service extension, requiring more sophisticated measures. Lastly, we did not collect more subjective evaluation data by survey, which might be useful for the interpretation of how participants evaluated brand extension. For instance, we could perform an in-depth interview or a review session with the think-aloud protocol.

To resolve these problems, advanced stimuli grouping methods should be developed. To our knowledge, little is known about the fit level of service brand extension. Because stimuli grouping method is a basic step for service-to-service brand extension, it will remain crucial for the follow-up studies. Additionally, we compared our results with previous studies where Chinese (Ma et al., 2007, 2008, 2010, 2014a,b; Wang et al., 2012; Shang et al., 2017) or Indo-European language speakers (Fudali-Czyż et al., 2016) participated in. Previous studies reported that a cultural difference could affect favorability in evaluating brand extension (Monga and John, 2007). Chinese, Korean, and Indo-European people have different characters, languages, and cultures. The difference between cognitive processes between previous studies and present study could be affected by those differences. Therefore, further studies about either service-to-service brand extension evaluation of participants in different cultural backgrounds or goods-to-goods brand extension evaluation of Korean participants will enhance understanding of cognitive process in evaluating brand extension.

Despite above limitations, our findings suggest that EEG analysis show important information that we cannot know only with behavioral data (i.e., reaction time) analysis. The reaction time analysis revealed that participants’ responses were significantly slow to mid-fit stimuli groups. However, low- and high-fit stimuli groups could not be distinguished only with reaction time data. In contrast, EEG result could distinguish those stimuli, suggesting two-stage cognitive process that first detecting and dropping low-fit stimuli based on improbability and second evaluating fit level based on incongruence. Therefore, a novel marketing tool to expect fit level of pre-formed brand extension before launching it using EEG analysis could be used for brand marketers. In addition, the present study suggested endogenous cognitive process in evaluating brand extension. For example, lower N400 amplitude and high P300 amplitude in low-fit stimuli indicate low integration and high improbability between parent brand and extended service, respectively. This result indicates that neuromarketing could help marketers, by providing objective and effective information about consumer decision making, in forming a marketing strategy as well as in evaluating pre-formed marketing strategy.

## Conclusion

The present study investigated neural responses during consumers’ evaluation of service-to-service brand extension. The analysis of ERPs revealed three components: N2, P300, and N400, which were different from those of the previous goods-to-goods brand extension studies. Based on behavioral responses regarding the fit level of parent brand and extended service, we divided the stimuli into three groups (low-, mid-, and high-fit groups) and compared each component between the groups. As a result, N2 did not show any significant difference between the fit groups, implying that different fit levels did not influence a basic perceptual process. P300 showed higher amplitudes for the low-fit group than others, indicating that participants might first sort out discrepant brand extension and then evaluated more congruent extension only. N400 showed more negative amplitudes for the mid- and high-fit groups, indicating facilitated semantic integration of extended service with parent brand for these groups. These neural responses suggest that the evaluation of service-to-service brand extension may involve different cognitive processes from those in the evaluation of goods-to-goods brand extension, and that different marketing strategies may be deployed for different types of brand extension.

## Author Contributions

TY participated in all aspects of the work, designed and conducted the experiment, analyzed the data, and wrote the manuscript. SL and ES conducted the experiment. S-PK oversaw the study and managed every part of research. All authors read and approved the final manuscript.

## Conflict of Interest Statement

The authors declare that the research was conducted in the absence of any commercial or financial relationships that could be construed as a potential conflict of interest.
