# Supplementary material for: Characteristics of Human Brain Activity during the Evaluation of Service-to-Service Brand Extension
Source: Front Hum Neurosci. 2018 Feb 9;12:44. doi: 10.3389/fnhum.2018.00044 (PMC5811480; doi:10.3389/fnhum.2018.00044)
Supplement: Supplementary file 1 [file Table_1.DOCX]

Supplementary Material

Characteristics of human brain activity during the evaluation of the service-to-service brand extension

Taeyang Yang, Seungji Lee, Seomoon Eunbi, Sung-Phil Kim*

*** Correspondence:** Corresponding Author: spkim@unist.ac.kr

Participants filled in the survey form (**Supplementary Table 1**) including following seven questions for each S1-S2 pair with the 7-Likert scale (1-7):

Q1: It is acceptable to extend *S1 brand* to *S2 service*.

Q2: *S2 service* of *S1 brand* is expected to have high-quality.

Q3: *S2 service* of *S1 brand* is preferred.

Q4: *S2 service* and main service of *S1 brand* are considered similar to each other.

Q5: This *S1-S2 brand extension* is positively considered.

Q6: This *S1-S2 brand extension* has a positive effect on *S1 brand*.

Q7: I have sufficient knowledge about *S2 service*.

Supplementary Table 1. The sample of the survey sheet used for the study. The actual survey sheet presented to participants was reformatted here according to Frontiers Word Templates for publication.

| **Parent service brand (S1)** | **Extension service (S2)** | **Q1** | **Q2** | **Q3** | **Q4** | **Q5** | **Q6** | **Q7** |
| --- | --- | --- | --- | --- | --- | --- | --- | --- |
| 11st | TV home shopping channel |  |  |  |  |  |  |  |
| 11st | Fashion magazine |  |  |  |  |  |  |  |
| 11st | Travel agency |  |  |  |  |  |  |  |
| 11st | Insurance service |  |  |  |  |  |  |  |
| 11st | Marketing consulting |  |  |  |  |  |  |  |
| 11st | Newspaper |  |  |  |  |  |  |  |
| 11st | Food delivery |  |  |  |  |  |  |  |
| G-market | TV home shopping channel |  |  |  |  |  |  |  |
| G-market | Fashion magazine |  |  |  |  |  |  |  |
| G-market | Travel agency |  |  |  |  |  |  |  |
| G-market | Insurance service |  |  |  |  |  |  |  |
| G-market | Marketing consulting |  |  |  |  |  |  |  |
| G-market | Newspaper |  |  |  |  |  |  |  |
| G-market | Food delivery |  |  |  |  |  |  |  |
| Kookmin bank | TV economy channel |  |  |  |  |  |  |  |
| Kookmin bank | Economy magazine |  |  |  |  |  |  |  |
| Kookmin bank | Education service |  |  |  |  |  |  |  |
| Kookmin bank | Legal counseling |  |  |  |  |  |  |  |
| Kookmin bank | Hospital |  |  |  |  |  |  |  |
| Kookmin bank | Simultaneous interpretation |  |  |  |  |  |  |  |
| Kookmin bank | Airline |  |  |  |  |  |  |  |
| Shinhan bank | TV economy channel |  |  |  |  |  |  |  |
| Shinhan bank | Economy magazine |  |  |  |  |  |  |  |
| Shinhan bank | Education service |  |  |  |  |  |  |  |
| Shinhan bank | Legal counseling |  |  |  |  |  |  |  |
| Shinhan bank | Hospital |  |  |  |  |  |  |  |
| Shinhan bank | Simultaneous interpretation |  |  |  |  |  |  |  |
| Shinhan bank | Airline |  |  |  |  |  |  |  |
| Korean air | Travel agency |  |  |  |  |  |  |  |
| Korean air | Travel information magazine |  |  |  |  |  |  |  |
| Korean air | Simultaneous interpretation |  |  |  |  |  |  |  |
| Korean air | Accommodation reservation |  |  |  |  |  |  |  |
| Korean air | TV documentary channel |  |  |  |  |  |  |  |
| Korean air | Rent-a-car |  |  |  |  |  |  |  |
| Korean air | Education |  |  |  |  |  |  |  |
| Asiana airline | Travel agency |  |  |  |  |  |  |  |
| Asiana airline | Travel information magazine |  |  |  |  |  |  |  |
| Asiana airline | Simultaneous interpretation |  |  |  |  |  |  |  |
| Asiana airline | Accommodation reservation |  |  |  |  |  |  |  |
| Asiana airline | TV documentary channel |  |  |  |  |  |  |  |
| Asiana airline | Rent-a-car |  |  |  |  |  |  |  |
| Asiana airline | Education |  |  |  |  |  |  |  |
| Lotte hotel | Club |  |  |  |  |  |  |  |
| Lotte hotel | Catering service |  |  |  |  |  |  |  |
| Lotte hotel | Travel information magazine |  |  |  |  |  |  |  |
| Lotte hotel | Travel agency |  |  |  |  |  |  |  |
| Lotte hotel | Legal counseling |  |  |  |  |  |  |  |
| Lotte hotel | Hospital |  |  |  |  |  |  |  |
| Lotte hotel | Finance |  |  |  |  |  |  |  |
| Warkerhill hotel | Club |  |  |  |  |  |  |  |
| Warkerhill hotel | Catering service |  |  |  |  |  |  |  |
| Warkerhill hotel | Travel information magazine |  |  |  |  |  |  |  |
| Warkerhill hotel | Travel agency |  |  |  |  |  |  |  |
| Warkerhill hotel | Legal counseling |  |  |  |  |  |  |  |
| Warkerhill hotel | Hospital |  |  |  |  |  |  |  |
| Warkerhill hotel | Finance |  |  |  |  |  |  |  |
